# Novel Approaches Guiding the Future of Spinal Biologics for Bone Regeneration

**DOI:** 10.1007/s12178-022-09757-4

**Published:** 2022-04-18

**Authors:** Eileen N. Phan, Wellington K. Hsu

**Affiliations:** 1 School of Medicine, University of California, Davis, 4610 X St, Sacramento, CA 95817 USA; 2 Simpson Querrey Institute (SQI), Northwestern University, 676 N. Saint Clair, Chicago, IL 60611 USA; 3 Department of Orthopaedic Surgery, Northwestern University, 676 N. Saint Clair, Chicago, IL 60611 USA

**Keywords:** Nanotechnology, Peptide amphiphiles, 3D printing, Additive manufacturing, Spine fusion, Bone regeneration

## Abstract

**Purpose of Review:**

Despite the continued growth of spine fusion procedures, the ideal material for bone regeneration remains unclear. Current bone graft substitutes and extenders in use such as exogenous BMP-2 or demineralized bone matrix and hydroxyapatite either have serious complications associated with use or lead to clinically significant rates of non-union. The introduction of nanotechnology and 3D printing to regenerative medicine facilitates the development of safer and more efficacious bone regenerative scaffolds that present solutions to these problems. Many researchers in orthopedics recognize the importance of lowering the dose of recombinant growth factors like BMP-2 to avoid the complications associated with its normal required supraphysiologic dosing to achieve high rates of fusion in spine surgery.

**Recent Findings:**

Recent iterations of bioactive scaffolds have moved towards peptide amphiphiles that bind endogenous osteoinductive growth factor sources at the site of implantation. These molecules have been shown to provide a highly fluid, natural mimetic of natural extracellular matrix to achieve 100% fusion rates at 10–100 times lower doses of BMP-2 relative to controls in pre-clinical animal posterolateral fusion models. Alternative approaches to bone regeneration include the combination of existing natural growth factor sources like human bone combined with bioactive, biocompatible components like hydroxyapatite using 3D-printing technologies. Their elastomeric, 3D-printed scaffolds demonstrate an optimal safety profile and high rates of fusion (~92%) in the rat posterolateral fusion model.

**Summary:**

Bioactive peptide amphiphiles and developments in 3D printing offer the promising future of a recombinant growth factor- free bone graft substitute with similar efficacy but improved safety profiles compared to existing bone graft substitutes.

## Introduction

From 2004 to 2015, elective lumbar fusion operations increased 62.3% from 122,679 to 199,140 cases per year [1]. In the context of this growth, the need for developing methods for reducing non-union rates to decrease the number of reoperations has become even more important. These innovations have occurred at multiple levels, including new structural implants, synthetic bone substitutes, and surgical techniques. Despite these improvements, however, there remains a void for a safe, reliable, and cost-effective bone graft substitute.

Iliac crest bone graft (ICBG), which has been utilized for over 50 years, is associated with complications such persistent pain and donor site morbidity postoperatively [2, 3]. Moreover, autografts like ICBG vary in available amount and quality depending on the patient(s) they are harvested from [4, 5]. Despite such associated costs, rates of non-union for ICBG can be up to 40% at 6 months and 20% at 12 months [6•]. While bone morphogenetic protein-2 (BMP-2) was considered a promising bone graft substitute, offering lower surgical morbidity, reduced blood loss, and reduced rates of non-union (~14% at 6 months and ~12% at 12 months) compared to ICBG, it has been associated with potentially serious complications [6•]. The use of BMP-2 spiked from 2005 to 2009 (28–31%) and then decreased in 2010 and 2011 (10–11%) in single-level posterior lumbar interbody fusions (PLIFs), possibly in association with case reports and reported side effect profiles [7]. Soon after its approval as an alternative to ICBG in anterior lumbar interbody fusion (ALIFs) in 2002 as INFUSE™ (recombinant human BMP-2 on ACS), several studies began reporting complications associated with its use including inflammation, radiculopathy, ectopic bone formation, osteolysis, subsidence, and urogenital events, as well as potential enhancement of tumor function associated with off-label use [8, 9]. These complications may be due to the requirement of supraphysiological dosing and exacerbated by the burst release of BMP-2 upon implantation when implanted on ACS, such as in INFUSE™ [10].

Future synthetics and biologics will need to address these concerns while achieving a high fusion rate. The ideal bone graft substitute should provide an osteoconductive scaffold, osteoinductivity, osteogenicity, and be easy for the surgeon to handle at a reasonable cost. This article will review emerging innovations and technologies that may lead to efficacious bone graft substitutes including peptide amphiphiles and additive manufacturing.

## Peptide Amphiphiles

**Fig. 1:**
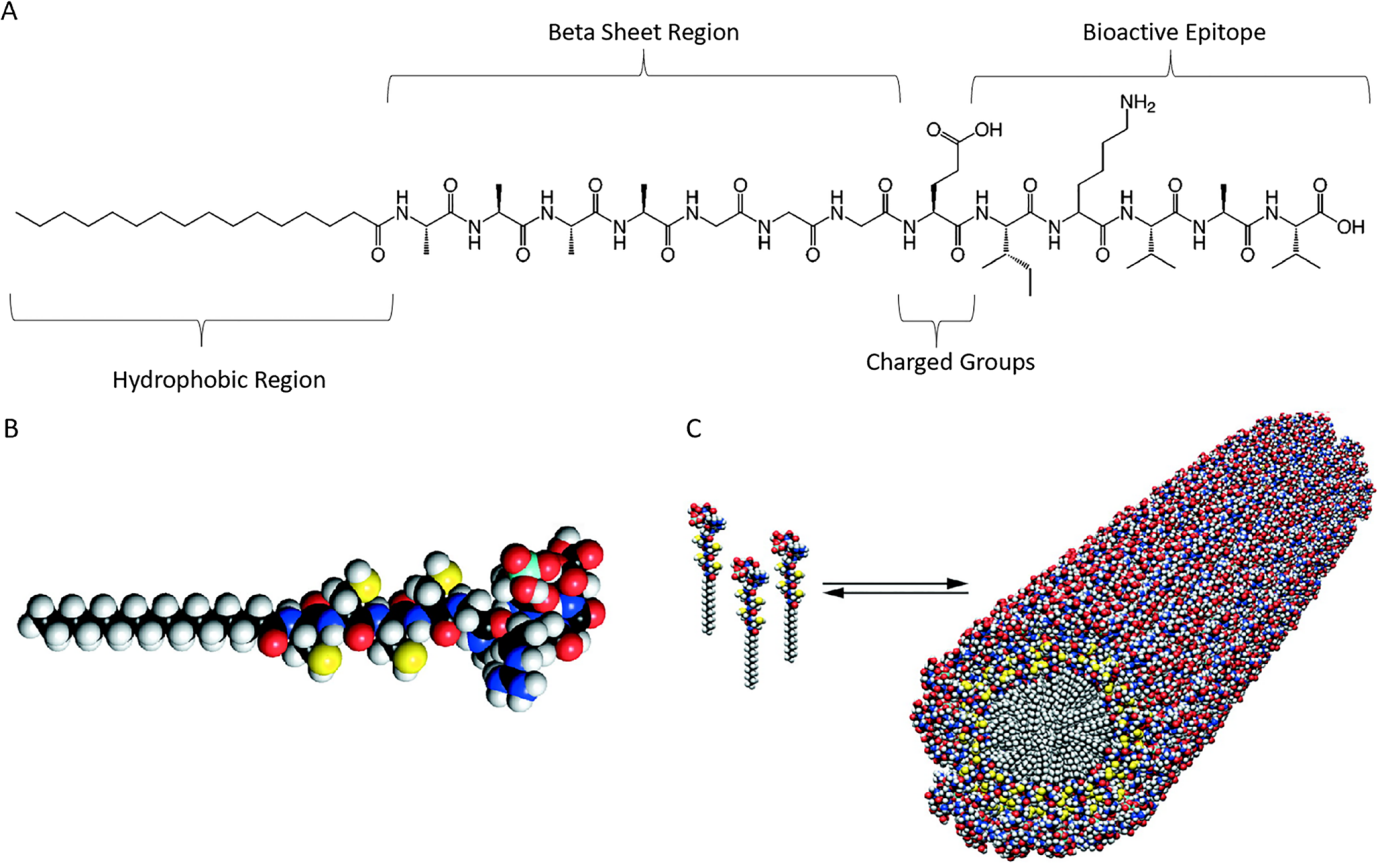
**a** Chemical structure of a representative peptide amphiphile with four domains. Reprinted (adapted) with permissions from [15]. Copyright (2003) American Chemical Society. **b-c** Molecular model of a representative peptide amphiphile and three-dimensional model of peptide amphiphiles selfarranging into nanofibers. From [16]. Reprinted with permission from AAAS

Peptide amphiphiles (PAs) are a broad class of molecules or building blocks composed of a mixture of hydrophobic and hydrophilic amino acids that self-assemble in aqueous solutions due to molecular forces including hydrogen bonds, dipole, and ion interactions [11–14] (Fig. 1). Based on their specific composition, these biomaterials are able to form sac-like structures, tubular fibers, or hydrogels [17–20]. The self-assembly process occurs based on triggers from pH, temperature, or exposure to salts [16, 21, 22]. PAs also have tunable mechanical properties, self-healing abilities, resistance to proteolysis, biodegradability, and biocompatibility [12, 19, 22–24]. Moreover, these structures can be chemically and biologically tailored to act as a conduit for drug delivery, tissue engineering, and regenerative medicine [24, 25]. Due to all these unique properties, PA-based biomaterials are an area of growing interest in bone graft therapies.

## Heparin or Heparan Sulfate–Binding PAs

One of the first systems for this use involved a heparin-binding PA (hbPA) capable of binding a variety of growth factors [26]. The hbPA system consisted of a short hydrophilic heparin-binding peptide conjugated to a hydrophobic alkyl chain that self-assembled into a close mimetic of natural extracellular matrix (ECM) [26]. Through the cell sandwich assay, which assesses angiogenic potential from the ability of the tested material to induce reorganization of sandwiched endothelial cells into tubular structures, the hbPA system potentiated both exogenous and endogenous angiogenic growth factors like fibroblast growth factor-2 (FGF-2) and vascular endothelial growth factor (VEGF) [26, 27••]. Interestingly, hbPA-heparin matrices without added growth factors would still form tubules, suggesting that they were able to use endogenous growth factors secreted from the cells of the assay. However, these tubules were smaller in number and appeared at later time points than hbPA-heparin matrices with added growth factors [26, 27••]. Although this system was first tested for angiogenic potential, the hbPA system was also targeted towards BMP-2 and thus osteogenic potentiation.

These hbPA-heparan sulfate nanofibers were utilized to regenerate bone in a rat critical size femoral defect model [28]. Compared to the control which required 11μg of BMP-2 to bridge the defect effectively, the hbPA-heparan sulfate nanofibers embedded within the pores of absorbable collagen sponge (ACS) achieved >50% bridging with only 1 μg of BMP-2 [28]. In vitro assessment of the hbPA showed enhanced BMP-2 retention, with individual contributions from both heparan sulfate and the nanofibers [28]. However, neither heparan sulfate nor nanofibers alone were as effective in achieving fusion compared to the combination, suggesting that certain properties of the biomimetic system beyond its individual components enhanced signaling efficiency [27••]. Unique properties of the system allowed for favorable ligand receptor binding within the free-flowing ECM mimetic, explaining why in the absence of peptide amphiphile nanofibers, heparan sulfate alone was incapable of promoting significant bone regeneration [27••, 29].

## BMP-2-Binding PAs

As a ubiquitously expressed glycosaminoglycan, heparan sulfate binds a variety of proteins and growth factors, most of which are non-specific to osteogenesis [30, 31]. To avoid the need of sourcing heparan sulfate, subsequently, a BMP-2-binding PA (BMP2b-PA) that would display a BMP-2-binding peptide epitope sequence on the surface of the nanofibers to bind BMP-2 specifically [32] was developed. Unlike natural heparan sulfate, bioactive epitopes are more stable and have a longer half-life [27•]. Several iterations of this BMP2b-PA were tested on C2C12 pre-myoblast cells, with heparin as a positive control, for assessment of osteogenic potential: diluted BMP2b-PA (equal parts by weight percentage of BMP2b-PA and diluent PA), BMP2b-PA, and diluent PA (BMP2b-PA without the BMP-2-binding epitope) [32]. Although all PA systems significantly increased ALP activity compared to BMP-2 alone, diluted BMP2b-PA exhibited the highest increase in ALP activity after 3 days of treatment [32]. Further assessment of the PA systems on changes in expression of osteogenic genes including Runx2, Osterix (Osx), and osteocalcin (OCN) mirrored ALP activity results [32]. Osteogenic mRNA levels from [50 ng∙mL^−1^] BMP-2 + [10 μg∙mL^−1^] of either diluted BMP2b-PA, BMP2b-PA, diluent PA, or heparin all led to enhanced osteoblastic differentiation relative to BMP-2 alone; diluted BMP2b-PA had the greatest increase in mRNA levels, significantly higher than either BMP2b-PA or diluent PA alone [32]. Both PA gels were found to retain BMP-2 more than collagen sponge after 28 days in vitro [32]. Diluted BMP2b-PA was also found to capture more BMP2 on a growth factor assay than diluent PA at 4 h, with no significant difference between the two after 16 h [32].

Strong in vitro success of the diluted BMP2b-PA led to in vivo testing in the rat posterolateral fusion (PLF) model. At 8 weeks post-treatment, diluted BMP2b-PA elicited the highest fusion scores [32]. Compared to collagen sponge which requires 10 μg of BMP-2, BMP2b-PA achieved a 100% fusion rate at 1 μg of BMP-2, allowing for a 10-fold reduction of required BMP-2 [32]. Remarkably, diluted BMP2b-PA alone, without the addition of any exogenous growth factor, produced a 42% fusion rate, suggesting that the endogenous growth factor in this biologic environment was adequate to fuse the spine in some animals [32].

## Heparin or Heparan Sulfate Mimetic PAs

**Fig. 2:**
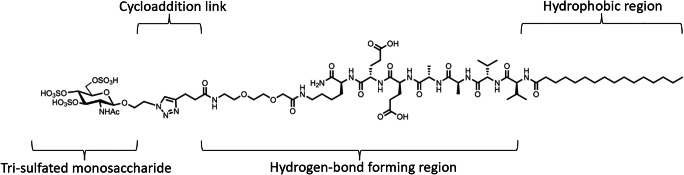
Chemical structure of the glycopeptide peptide amphiphile, consisting of a peptide amphiphile (with hydrophobic and hydrogen-bond forming domains) conjugated to a bioactive tri-sulfated 3,4,6S-N-acetyl glucosamine via cycloaddition. Adapted with permissions from Nature Nanotechnology [35]

Since the bone is a highly vascularized structure, in addition to BMP-2, angiogenic growth factors such as VEGF and FGF are also important to the bone regeneration process [33, 34]. Motivated by the idea of capturing these other endogenous growth factors important to the bone healing process, a tri-sulfated monosaccharide attached to a PA molecule known as a glycopeptide PA (GPA) [35] was designed (Fig. 2). Sulfation is the structural hallmark of the natural polysaccharides’ ability to bind hundreds of proteins in biology [36]. Similar to previous PA iterations, the non-covalent bonds within GPA supramolecular structures could dynamically rearrange, freeing the tri-sulfated monosaccharides to access different configurations and adapt to the heparin-binding domains of different proteins [37]. As such, this GPA system was able to bind five biologically important proteins in cell development: BMP-2, BMP-4, FGF-1, FGF-2, and VEGF [35]. Similar to hbPA, GPA was found to potentiate BMP-2 signal based on the inherent bioactivity of the PA assembly and not from bound heparan sulfate [35]. Culture of C2C12 myoblast cells with 75 ng/mL of BMP-2 in the presence of heparin, heparan sulfate, or GPA found that heparan sulfate enhanced ALP expression by 3×, heparin by 5×, and GPA by 9× compared to BMP-2 alone [35]. The GPA system promoted higher mRNA expressions of ALP and OCN relative to heparin or saline controls [35]. The GPA system also performed better on mineralization assays than growth factor alone, while other control nanostructures had minimal effect [35]. Although the exact binding mechanisms of GPA and growth factors are unclear, GPA enhanced signaling of wild-type BMP-2 but not of a mutant BMP-2 lacking its heparin-binding domain, which suggests that the ability to potentiate signaling stems from its ability to mimic heparin and heparan sulfate [27, 35]. The GPA was further tested in vivo by absorption into porous collagen sponges, where the biomaterial was able to achieve successful spine fusion in the pre-clinical rat PLF model [35]. In this study, 100% fusion was achieved at 100 times a lower dose of BMP-2 in animals with GPA at an extremely low dose of 0.1 μg/rat compared to collagen sponge control animals [35].

Importantly, lowering the required dose of BMP-2 for successful fusion would reduce the associated side effects of rhBMP-2 and allow for safer spine surgery. Although this scaffold shows great promise as an osteoconductive, osteoinductive, and osteogenic biocompatible material for spine fusion that can reduce the required effective dose of BMP-2, future directions include the development of a collagen-glycosylated PA (GPA) scaffold to not only reduce but eliminate the need for BMP-2.

## Additive Manufacturing

The ideal scaffold for spine fusion must be biocompatible, biodegradable, osteoconductive, osteoinductive, and of appropriate size with interconnected pores to provide an optimal environment for the bone remodeling process [38]. Additive manufacturing (AM) or 3D printing brings the field of tissue engineering closer to this goal by facilitating a fast and precise system of customizing artificial scaffolds [39]. The AM process is highly flexible, capable of creating structures from a wide host of materials including polymers, composite biomaterials, and ceramics [41]. As such, AM has been widely incorporated in the bioprinting of dermal layers, cartilage, bone defect, and surgical implants [39]. Compared to traditional methods of scaffold engineering that experienced difficulties in controlling the porosity and exact structure of the scaffold, the use of 3D printing allows for precision and reproducibility of design [39, 40].

3D printing relies on computer-aided design (CAD), where (1) a 2D image that is converted to a 3D model or (2) a representation of an existing 3D entity is stored as a stereolithographic (.STL) file [39]. The file is then converted to a coding file that provides commands for printer outputs including speed, movement specifications, and ink distributions to build the structure from the bottom up, layer by layer, allowing for individual customization of each section and slice of product [39].

**Fig. 3:**
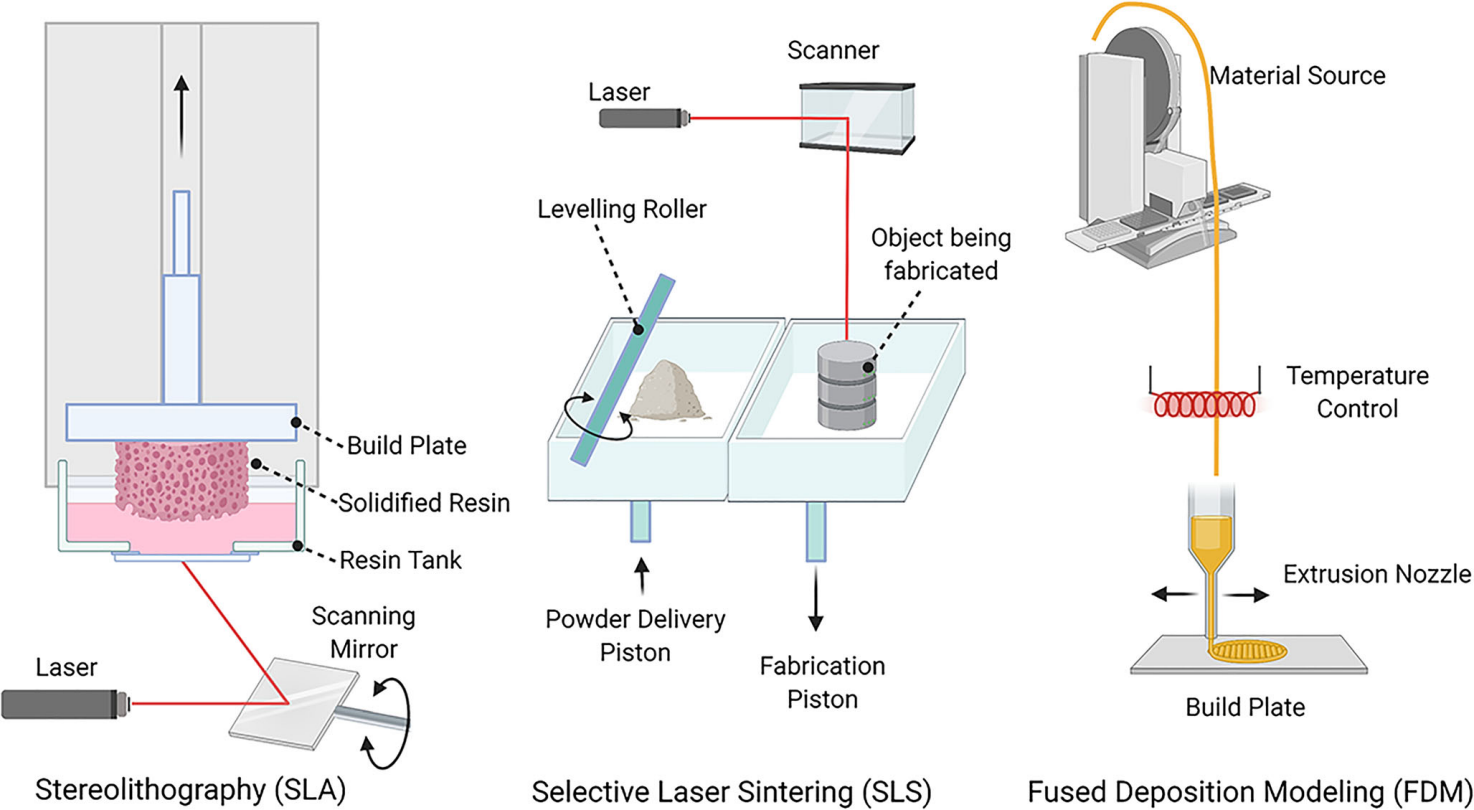
Representations of stereolithography (SLA), selective laser sintering (SLS), and fused deposition modeling (FDM) created with publishing permissions through BioRender.com

Based on printer assembly, raw material requirements, and manufacturing technique, 3D printers can be broadly categorized into 3 groups: stereolithography (SLA), selective laser sintering (SLS), and fused deposition modeling (FDM) [41] (Fig. 3). SLA was developed first in the early 1980s by Charles Hull who created a layer-by-layer model from a CAD by photopolymerization of a UV-curable material [39, 42]. However, neither SLA nor SLS is ideal for biological tissue engineering which is limited to UV-curable polymers which are not typically biocompatible or biodegradable [43•]. SLS requires post-printing processing to remove excess and unfused powders, which can be difficult and extremely cumbersome, especially if the pores in the scaffold structure are in the micron range as with bone [39, 43•]. Moreover, the high intensity of applied laser generally degrades the polymer starting material [43•]. Similarly, FDM, which is the method of melting materials and fusing them together to 3D print, can degrade structure components due to required extrusion at high temperatures [39, 44]. However, FDM works well with several thermoplastic, biocompatible polymers including poly D,L-lactic-co-glycolic acid (PLGA), polyether ether ketone (PEEK), and polycaprolactone (PCL) [45–48]. PCL is an aliphatic polyester with good processing and tunable mechanical properties, making it one of the most versatile scaffolding materials for bone implants. PCL biodegrades on the order of years compared to PLGA which is on the order of weeks to months. Depending on the requirements of the bone scaffold, PLGA, which also allows for controllable mechanical properties with earlier biodegradability, may be more effective and practical [49, 50].

A special evolution of FDM, called Extrusion-Based (EB) 3D printing, allows for the incorporation of bioactive substances including growth factors, proteins, drugs, and cells into the scaffold structure [50••]. This allows for the construction of scaffolds that are more than biocompatible simple support structures. It provides the opportunity for bioactive scaffolds that directly encourage bone growth at the area of defect. In this context, both demineralized bone matrix (DBM) and the ceramic hydroxyapatite (HA) are appropriate additives, each valued for their separate properties and contributions to the bone regeneration process.

DBM is a cost-effective, readily available form of allograft created from an acid extraction process of cadaveric bone. However, the preparation process removes many mineral components, leaving leaves a particular powder mixture of type I collagen and non-collagenous proteins, such as BMP [4]. Due to its collagen components, DBM provides a great osteoconductive surface. However, its osteoinductive factors such as BMP, TGF-β, and PDGF only make up 5% of its final composition, making DBM weakly osteoinductive [4]. Traditional carriers of DBM including glycerol, poloxamer, gelatin, calcium sulfate, lecithin, and hyaluronic acid are bio-inert, and further dilute the amount of native growth factors in DBM [51]. Moreover, the amount of osteogenic activity is highly dependent on the type of bone used and its preparation process. Certain preparations of DBM can be very acidic, such as when it is combined with a glycerol carrier, which may have detrimental effects on host cells in large quantities [4]. Some DBM-based products can also have poor handling characteristics leading to implant migration and subsequent heterotopic bone formation. Finally, there is significant variability in spine fusion rates (63–89%) among different types of DBM preparations with autograft [52]. Newer scaffold developments of DBM should focus on consistently enhancing its osteoinductivity as well as surgical handling properties.

HA is composed of calcium phosphate and naturally occurs in biological systems as the primary inorganic component of normal bone [53]. Like DBM, HA has a long history of usage as a bone graft extender due to its high biocompatibility, strong osteoconductivity, and easy osteointegration [53]. Moreover, HA provides a stable surface for osteoblastic cell adhesion, growth, and differentiation [53]. However, these properties of HA are highly dependent on its porosity, with many studies demonstrating the necessity of hierarchical (macro- and micro-) porosity to successful bone regeneration [54, 55••, 56, 57••]. Macro-porosity (pore sizes 100–500 μm) provides the space for cell colonization and blood vessel formation [56, 57••] while micro-porosity (pore sizes <10 μm) provides surface roughness for cell attachment and protein absorption [56, 57••].

While 3D printing addresses the concern of consistency of scaffold design and quality in both DBM- and HA-based scaffolds, the true advantage of additive manufacturing comes in the ability to merge these materials together in synergistic combination therapies. Using extrusion-based 3D printing at low temperatures, a HA-DBM scaffold that had elastomeric properties and reduced brittleness was developed with macro-pores (500 μm) distributed through the material [58••]. This porous, elastomeric, HA-DBM composite scaffold achieved a fusion rate of 92% in the pre-clinical rat PLF model without the use of recombinant growth factor [58••]. Importantly, this rate was greater than 3D-printed HA only scaffolds (58%) and more than twice that of 3D-printed DBM scaffold (42%) [58••]. Moreover, de novo bone-like spicules were present only with HA and DBM together, and not HA or DBM alone, demonstrating the synergistic effect of HA and DBM on bone regeneration [58••].

From a safety perspective, compared to INFUSE™ (BMP-2 on ACS), the HA-DBM composite scaffold did not induce a significant host immune response [59••]. While BMP-2 treatment in vivo resulted in a significant fluid collection, HA-DBM treatment had no such inflammatory effect. Moreover, at 2 days post-operative, BMP-2-treated animals had higher circulating levels of inflammatory markers including IL-18, TNF-α, and MCP-1. At 5 days post-operative, IL-18 remained significantly elevated in BMP-2-treated animals. Remarkably, in contrast, HA-DBM treatment resulted in no significant elevation in cytokine levels relative to the ACS negative control group. Both BMP-2- and HA-DBM-treated spines showed a reduced range-of-motion relative to non-operated control specimens, demonstrating significant stabilization at the L4-L5 level. Overall, this elastomeric HA-DBM composite scaffold shows promise for promoting successful spine fusion in a rat model, with similar unilateral fusion rates to BMP-2 and an improved safety profile. Future iterations of this composite scaffold are working on controlling micropore size to fully capture the osteoinductive growth factors provided by DBM as well as any endogenous growth factors present at the site of regeneration.

## Conclusion

Nanotechnology and 3D printing are taking an increasingly important role in the development of new biological materials in spine fusion. The availability of these new synthetic methodologies in the form of peptide amphiphiles and additive manufacturing allows for the functionalizing of safe, existing growth factor sources such as DBM or endogenous growth factors present at the site of regeneration to provide better signals for bone remodeling. Although these scaffolds have demonstrated great potential in reducing the use of BMP-2 in cell and animal models, the complexity of the optimization process leaves much room for continual work until they may be applied in clinical trials. Results so far, however, are promising of a future recombinant growth factor-free bone graft substitute with similar efficacy but improved safety profiles compared to BMP-2.
